# Occupational heat stress assessment and protective strategies in the context of climate change

**DOI:** 10.1007/s00484-017-1352-y

**Published:** 2017-04-25

**Authors:** Chuansi Gao, Kalev Kuklane, Per-Olof Östergren, Tord Kjellstrom

**Affiliations:** 1 Thermal Environment Laboratory, Division of Ergonomics and Aerosol Technology, Department of Design Sciences, Faculty of Engineering, Lund University, Lund, Sweden; 2 Social Medicine and Global Health, Department of Clinical Sciences, Lund University, Malmö, Sweden; 3 Centre for Technology Research and Innovation (CETRI Ltd), Lemesos, Cyprus

**Keywords:** Global warming, Meteorological data, Occupational and environmental health, Heat stress index, Heat strain, Protection

## Abstract

Global warming will unquestionably increase the impact of heat on individuals who work in already hot workplaces in hot climate areas. The increasing prevalence of this environmental health risk requires the improvement of assessment methods linked to meteorological data. Such new methods will help to reveal the size of the problem and design appropriate interventions at individual, workplace and societal level. The evaluation of occupational heat stress requires measurement of four thermal climate factors (air temperature, humidity, air velocity and heat radiation); available weather station data may serve this purpose. However, the use of meteorological data for occupational heat stress assessment is limited because weather stations do not traditionally and directly measure some important climate factors, e.g. solar radiation. In addition, local workplace environmental conditions such as local heat sources, metabolic heat production within the human body, and clothing properties, all affect the exchange of heat between the body and the environment. A robust occupational heat stress index should properly address all these factors. This article reviews and highlights a number of selected heat stress indices, indicating their advantages and disadvantages in relation to meteorological data, local workplace environments, body heat production and the use of protective clothing. These heat stress and heat strain indices include Wet Bulb Globe Temperature, Discomfort Index, Predicted Heat Strain index, and Universal Thermal Climate Index. In some cases, individuals may be monitored for heat strain through physiological measurements and medical supervision prior to and during exposure. Relevant protective and preventive strategies for alleviating heat strain are also reviewed and proposed.

## Introduction

Global warming will unquestionably increase the impact of heat on individuals who work in already hot workplaces in hot climate areas (Smith et al. 2014; Błażejczyk et al. 2014; Spector and Sheffield 2014). The increasing prevalence of this environmental health risk will become a threat to public and occupational health in some areas of the world (Kjellstrom et al. 2009; Lundgren et al. 2013; Błażejczyk et al. 2014; Spector and Sheffield 2014), and is likely to reduce productivity in areas where an increasing proportion of the population could be pushed into poverty as a consequence (Kjellstrom et al. in press).

The concept of occupational heat stress refers usually to local workplace heat stress. In order to evaluate occupational heat stress, four thermal climate factors (air temperature, humidity, air velocity and heat radiation) should be measured; available weather station data may serve this purpose. Yet the use of meteorological data for the assessment of occupational heat stress in the context of climate change, faces challenges because not all the important climate factors (e.g. solar radiation in W/m^2^) have traditionally been directly measured in all weather stations (Parsons 2013). The measurements at a weather station usually include air temperature, barometric pressure, humidity, wind speed, wind direction, and precipitation. Moreover, it is not straightforward to translate outdoor measurements into the assessment of indoor workplace conditions, as indoor environments can be different (de Dear and Brager 1998; Błażejczyk et al. 2014; Yang et al. 2014). For example, weather stations are placed as distant as possible from any obstacles and local heat sources (e.g. workplace heat) and air velocity is usually measured at the standard height of 10 m. In addition, internal metabolic heat production in the body (physical work intensity), and clothing (e.g. protective clothing) impact on the exchange and balance of body heat with the external environment. Therefore, there is an urgent need to improve assessment methods linked to meteorological data for estimating the size of the problem and for developing appropriate interventions at individual, workplace and societal level (Kjellstrom and McMichael 2013).

Any comprehensive thermal stress index will need to consider all the six factors outlined for appropriate assessment of occupational heat stress (Parsons 2014; Epstein and Moran 2006). Two clothing properties, thermal insulation and evaporative resistance, are important factors affecting human body heat exchange in hot environments. Outdoor work in a hot climate imposes high heat stress risks due to strenuous work and solar radiation. There is further increased risk if there is any deterioration of influential risk factors such as (1) local increases in mean air temperatures and/or frequencies of heat events, (2) lack of protective measures such as shade, fans, air-conditioning, or other adaptation approaches, (3) more intense physical work demands and/or fewer rest breaks, and (4) workforces that are more susceptible to heat such as un-acclimatized, untrained and frail workers (Parsons 2014; Spector and Sheffield 2014; Błażejczyk et al. 2014).

## Occupational heat stress evaluation indices in the context of climate change

A comprehensive register of 162 human thermal climate indices has been recently assembled and categorized into eight classes by de Freitas and Grigorieva (2015). The development of a heat stress index as “a single value that integrates the effects of the basic parameters in any human thermal environment such that its value will vary with the thermal strain experienced by the individual” (Epstein and Moran 2006) has been fraught with difficulties. A number of scientists have approached the issue in different ways. Epstein and Moran (2006) summarized 46 indices that have been published since Haldane (1905) proposed the “Wet-bulb Temperature” more than 100 years ago. This single parameter is still used in climate change impact research. Sherwood and Huber (2010) and Pal and Eltahir (2015) used wet bulb temperature (psychrometric) T_w_ measured in well ventilated conditions for the assessment of survivability of humans due to heat stress in hot climates. Based on physics and heat transfer theory, but without any physiological considerations, a psychrometric wet bulb temperature of 35 °C was considered a limit for survival, when only resting metabolic heat production was taken into consideration. Clothing and solar heat radiation were not considered for this evaluation.

Havenith and Fiala (2016) recently reviewed 35 heat stress indices and models, and pointed out that simple indices are most popular for use in the field. The acceptance of complex models seems limited and is usually used in a research context. No index meets all and sometimes conflicting demands of simplicity, availability, accuracy, validity, reliability, repeatability, continuous recording, data storage, etc. Different heat stress indices have their own advantages and shortcomings. While there is debate about which index should be used, an appropriate choice is probably dependent on conditions, user’s previous experience, possibility to compare results between studies and the level required for heat stress assessment (screening, analysis, etc.). At present, based on international standardization work (ISO), Wet Bulb Globe Temperature (WBGT) is meant to be used for heat stress screening (ISO 7243) and Predicted Heat Strain (PHS) for assessments of both heat stress and strain (ISO 7933), forming the base for heat stress management.

There are several dimensions to the evaluation of heat stress, which are not always clearly described in the background to different indices. These include:

1. the difference between **occupational (working people) and general population heat stress** evaluation: workplaces have special features that may increase heat exposure, and working people including people who are doing exercises and leisure activities often have a higher metabolic rate than people who are not working, which influences the heat stress;
2. **individual assessments vs population based assessments**: a focus on a population rather than on individuals demands assumptions to be made about population characteristics (e.g. age, gender, body weight and height, variability, vulnerability, acclimatization), workplace clothing and activities (e.g. metabolic rate);
3. underlying **evaluation principles of heat stress**: rational indices based on physics (heat transfer and heat balance), empirical indices based on objective and subjective heat strain: thermal physiological responses (e.g. core body and skin temperatures, sweat rate, dehydration) or psychological responses (perceived thermal sensation and comfort) and related physical and cognitive performance (productivity) reduction; direct indices based on direct measurements of environmental variables.
4. assessments of **current heat stress or estimates of future heat stress**: climate change impact assessments deal with future levels and therefore calculations need some assumptions about local environment characteristics, protective clothing and work intensity to estimate workplace heat stress levels. In addition, the heat stress index needs to be reasonably accurate up to the heat levels that may occur in the future;
5. the difference between the **calculated index value and the limit value** in the standards and guidelines: a calculated heat stress index may be a meaningful indicator of potential heat impacts, but standards that limit work activities and/or exposure time, are also influenced by the properties of clothing and work intensity; individual authorities can set different heat exposure limits.

Some assumptions (e.g. clothing and work intensity) at population level need to be made for heat stress assessments, based on climate models. We present the evaluation method of thermal strain and five heat stress indices and heat stress assessment methods with a focus on their applications in workplace situations: Individual heat strain monitoring, Wet Bulb Globe Temperature (WBGT), Discomfort Index (DI), Predicted Heat Strain (PHS), and Universal Thermal Climate Index (UTCI) (2009). The latter is not developed for use in workplace situations, but we include it here as it is one of the most recent indices, and it has been promoted as an advanced heat stress index based on the meteorological input (Jendritzky et al. 2012; Blazejczyk et al. 2012).

### Individual heat strain monitoring

For the most detailed monitoring of individual thermal physiological responses to heat, personal measurements of core body (usually rectal) and skin temperatures, body mass loss (dehydration) due to sweating and heart rate can be carried out (ISO 9886: 2004). Furthermore, the measurement or estimation of oxygen uptake (VO_2_) quantifies aerobic workload and metabolic heat production of the whole body (ISO 8996: 2004). The rating of perceived thermal sensation and thermal comfort (ISO 10551: 1995; ISO 7730: 2005), and perceived physical exertion (Borg 1982), can complement the key quantitative measurements. Individual heat strain monitoring can detect early thermal physiological and psychological responses in vulnerable workers, (e.g. un-acclimatized, untrained, frail, ageing workers and workers with chronic diseases and disabilities), accordingly to provide timely and targeted personal protection and health care when confronted with heat waves (Parsons 2013; Gao et al. 2012; Zhao et al. 2013a, b). Due to biological variability it is not easy to accurately predict the response of any particular individual to climatic extremes. Therefore, it is necessary to provide appropriate medical supervision for individuals prior to and during severe heat stress exposures (ISO 12894: 2001).

### Wet bulb globe temperature (WBGT)

WBGT is among the most widely used occupational heat stress indices across the world (Parsons 2014; Havenith and Fiala 2016) and its inclusion in international (ISO 7243) and national (ACGIH 2009) standards indicates that it has been widely accepted since it was developed in the 1950s by the US Army (Yaglou and Minard 1957). The WBGT index can function as a screening tool for the assessment of heat stress (Parsons 2013; Epstein and Moran 2006). It applies to the evaluation of the mean effect of heat on humans during an eight-hour work day and during an hourly work period in determining work rest cycles, and the WBGT reference values do not apply to the evaluation of heat stress suffered during very short periods (Parsons 2013). WBGT can be measured in hot environments with and without solar radiation. According to the standard specifications (ISO 7243), certain types of measurement devices (e.g. a 150 mm black globe) should be used (Parsons 2013; Havenith and Fiala 2016). However, these devices were made in the 1950s so some improvements should be considered, e.g. allowing quicker response time (Johansson et al. 2017, in this issue). A revision of the standard (ISO 7243) has been in process for more than 5 years, and it is not clear whether the measurement devices will be updated. Several mathematical methods to calculate WBGT from weather station data have been published (Blazejczyk et al. 2012; Lemke and Kjellstrom 2012) and are of value for the assessment of climate change impact, particularly when there is lack of the standard measurement devices and lack of first-hand data measured in workplaces.

Based on the standard methods, WBGT can be calculated inside and outside buildings without solar radiation with these variables:

1 $$ \mathrm{WBGT}=0.7{\mathrm{T}}_{\mathrm{nw}}+0.3{\mathrm{T}}_{\mathrm{g}} $$

The formula is modified for measurements of the environment outside buildings with solar radiation:

2 $$ \mathrm{WBGT}=0.7{\mathrm{T}}_{\mathrm{nw}}+0.2{\mathrm{T}}_{\mathrm{g}}+0.1{\mathrm{T}}_{\mathrm{a}} $$

where:

T_nw_ is the natural wet bulb temperature (measured with a wet wick thermometer exposed to the local air movement and radiation), T_g_ is the temperature in the centre of a 150 mm diameter black globe, T_a_ is the air temperature shielded from radiation, but not restricted for air circulation around the sensor. Although wind speed is not directly measured and included in the above equations, T_nw_ and T_g_ combines the effects of radiation, humidity, air temperature and wind (Havenith and Fiala 2016).

An important advantage with WBGT as an occupational heat stress index in climate change impact assessments, is the availability of a small number of occupational epidemiology studies of heat stress on work capacity loss that was assessed and associated with WBGT levels (Wyndham 1969; Sahu et al. 2013). In spite of the availability of a large number of heat stress indices for many decades, practical field studies quantifying the relationship between heat stress indices and occupational illnesses/injuries are lacking. Most of the studies were purely descriptive and used air temperature as the only heat stress parameter (Xiang et al. 2014; Havenith and Fiala 2016). Furthermore, many epidemiological studies failed to include and report on other relevant factors for human heat exchange with the environment, such as metabolic rate and clothing.

The widespread use of WBGT in workplaces in many countries indicates that it is a valuable screening tool for workers and employers (Parsons 2014). However, WBGT has been criticized for being outdated after 60 years of use. d’Ambrosio Alfano et al. (2014) pointed out that the WBGT has, for instance, limited use in high humidity low air movement environments. They concluded that the WBGT index only considers the increase of core temperature, but does not consider the risk of dehydration due to excessive sweating, one of the main criteria for limiting the exposure in moderately hot work environments. In order to overcome such limitations, it may be necessary to use more than one heat stress index.

Lemke and Kjellstrom (2012) aimed at identifying a valid method to calculate current and future heat stress in hot areas of the planet using weather station data. However, neither T_nw_ nor T_g_ are directly measured in weather stations. Based on the analysis and comparative measurements of a number of available methods, Lemke and Kjellstrom (2012) recommended the method of Liljegren et al. (2008) for calculating outdoor WBGT in the sun and the method by Bernard and Pourmoghani (1999) for indoor or in full shade WBGT when estimating climate change impacts on heat stress at a population level. WBGT (T_g_ and T_nw_) estimated based on a simplified equation using meteorological parameters was compared with Universal Thermal Climate Index - UTCI (Błażejczyk et al. 2014). The results showed that the correlation was weak (Blazejczyk et al. 2012).

Solar radiation is not always directly measured and included in standard meteorological data. To accurately measure mean radiant temperature (T_mrt_) requires measurements of short- and long-wave radiation, diffuse and reflected radiation from six directions using pyranometer and pyrgeometers (Matzarakis et al. 2007; Schreier et al. 2013). However, not only T_mrt_ but also T_g_, T_nw_ can be indirectly estimated using various models based weather station data, geographical locations, time of the day, day of the year, etc. (Matzarakis et al. 2007; Lindberg et al. 2008; Schreier et al. 2013; Błażejczyk et al. 2014; Kruger et al. 2014). Estimates from a number of Asian cities (Kjellstrom et al. 2013) show that generally the afternoon WBGT values in the sun are 2–3 °C higher than the values in the shade. Another limitation that applies to all heat stress index estimates based on weather station data is that specific workplace conditions and urban environments can be significantly different from conditions at the nearby meteorological station (e.g. air velocity measured at 10-m height is different compared to that at the workplaces and in urban environments). These limitations caused by spatial differentiation and local heat sources (e.g. a smelter) when using meteorological data to estimate workplace WBGT are further reiterated by Spector and Sheffield (2014).

Current WBGT index reference values (ISO 7243) have considered physical activity and acclimatization. But the values can only apply to standard clothing (thermal insulation 0.6 clo, cotton and permeable) and various protective clothing options need to be considered. In addition, there is the specific sensitivity of a vulnerable workforce, e.g. older workers. A correction factor for different protective clothing properties is being considered in the revision of the standard based on related research (Bernard et al. 2007; Ashley et al. 2008). The working group responsible for developing the standard (ISO TC 159 SC5 WG1) has undertaken a review and a revision of the standard is ongoing. Parsons (2013) emphasized that the WBGT index will play a major role in monitoring occupational heat stress in the future. With climate change, occupational heat stress assessment using WBGT will be an important first line of defence in the avoidance of heat casualties (Parsons 2013), although the WBGT is limited in its applicability across a broad range of climatic scenarios due to the inconvenience of measuring T_g_ (Blazejczyk et al. 2012). Nevertheless, T_g_, T_nw_ and WBGT can be estimated based on standard weather station variables as discussed above.

### Discomfort index (DI)

3 $$ \mathrm{DI}=0.5{\mathrm{T}}_{\mathrm{w}}+0.5{\mathrm{T}}_{\mathrm{a}} $$

Where T_w_ is aspirated (psychrometric) wet-bulb temperature, and T_a_ is the air temperature.

Epstein and Moran (2006) pointed out that in many circumstances directly measuring the globe temperature T_g_ is cumbersome and impractical although they acknowledged the wide use of WBGT. Based on their review of 46 heat stress indices in literature and from their experience in Israel, they recommended a more simple and easily used index, Discomfort Index (DI), as a heat stress index while recognizing the lack of the integration of all six factors, for instance lack of heat radiation data. Clothing is only restricted to light summer clothing. Nevertheless, the authors found that DI correlates well with WBGT (R^2^ = 0.95 in the range of 15–33 °C of WBGT index, *n* = 108). However, T_w_ is not directly available from weather station data. Since T_w_ is a function of air temperature and relative humidity (RH%), it can be estimated using the following equation (Stull 2011)

4 $$ {\mathrm{T}}_{\mathrm{w}}={\mathrm{T}}_{\mathrm{a}}\kern.2em \mathrm{atan}\left[0.151977{\left(\mathrm{RH}\%+8.313659\right)}^{1/2}\right]+\mathrm{atan}\left({\mathrm{T}}_{\mathrm{a}}+\mathrm{RH}\%\right)-\mathrm{atan}\left(\mathrm{RH}\%-1.676331\right)+0.00391838{\left(\mathrm{RH}\%\right)}^{3/2}\kern.2em \mathrm{atan}\left(0.023101\mathrm{RH}\%\right){\textstyle \hbox{-} }4.686035 $$

### Predicted heat strain (PHS)

Since WBGT mainly works as a screening tool for occupational heat stress, another international standard - ISO 7933 (2004) *Ergonomics of the thermal environment − Analytical determination and interpretation of heat stress using calculation of the predicted heat strain (PHS)* can be used for detailed assessment (Parsons 2013). In contrast to WBGT, PHS describes a method based on human body heat balance equations for predicting both sweat rate and internal core temperature that the human body will develop as a result of heat stress. The heat balance is calculated by taking into account all factors involved in the heat transfer between the body and environment, i.e. four thermal climate factors, physical work intensity (metabolic rate) and clothing thermal properties (Malchaire et al. 2000, 2001; Malchaire 2006; Havenith and Fiala 2016). Methods for estimating metabolic rate and clothing insulation can be found in international standards ISO 8996 and ISO 9920 (2007), respectively. Lately, the thermal insulation of non-western clothing, including the commonly used clothing for indoor environments in Africa and Asia, has been added to the database (Havenith et al. 2015).

Currently the PHS method may be considered the most developed analytical method for predicting potential health problems for individuals due to work in the heat (Parsons 2013). However, the clothing insulation in the PHS model is limited to 1.0 clo. Neither the WBGT nor the PHS methods are valid for the assessment of rapidly changing environments and short exposure durations (Parsons 2013). The validity of the PHS model and possible integration with meteorological data in the context of climate change need further development and evaluation. Some limitations of the PHS model, when dealing with protective clothing (insulation and evaporative resistance) used in hot environments, can be found in recent studies (Wang et al. 2011, 2013; d’Ambrosio Alfano et al. 2015). In spite of the criticisms, the model is a useful tool for heat strain estimation (Kampmann et al. 2012; Rowlinson and Jia 2013; Kuklane et al. 2014, 2015; Lundgren et al. 2014). Calculations are available in the standard (ISO 7933) and on the website, e.g. www.eat.lth.se/termisk-miljoe/english/tools/. However, any web based calculation tools, if not directly promoted by related standard, should be used with caution.

### Universal thermal climate index (UTCI)

A recently proposed Universal Thermal Climate Index (UTCI) (2009), based on an advanced human thermoregulation model, directly uses meteorological data to predict the impact of outdoor climate on thermal physiological and perceptual responses (Jendritzky et al. 2012; Bröde et al. 2012a, b; Fiala et al. 2012; Havenith et al. 2012; Kampmann et al. 2012; Parsons 2013; Havenith and Fiala 2016). The UTCI equivalent temperature (°C) provides a one-dimensional characteristic of complex thermal environments as determined by air temperature, heat radiation, humidity and wind speed. The UTCI equivalent temperature for a given combination of wind, radiation, humidity and air temperature is defined as the air temperature of a reference environment, which produces the same heat strain. The reference environment is defined as an environment with 50% relative humidity (but water vapour pressure not exceeding 2 kPa; this will be reached at 29 °C), still air (v_a_ = 0.5 m/s at 10 m height, about 0.3 m/s at 1.1 m height) and mean radiant temperature equal to air temperature (Bröde et al. 2012a; Havenith and Fiala 2016). Mean radiant temperature is usually not directly measured at weather stations so it cannot be directly used as an input parameter for the calculation of UTCI, however, it can be estimated based on synoptic observations (e.g. cloud cover, global radiation) and various models based meteorological variables (Matzarakis et al. 2007; Lindberg et al. 2008; Schreier et al. 2013; Błażejczyk et al. 2014; Kruger et al. 2014). However, the application of UTCI to the assessment of outdoor environmental and occupational heat stress and resulting body heat strain, is limited by the assumed moderate activity level (135 W/m^2^) and the chosen exposure time of two hours (Bröde et al. 2016).

The assessment of the thermo-physiological effects of the atmospheric environment is one of the key issues in human biometeorology (Jendritzky et al. 2012). The UTCI provides an automated approach to incorporate complex physiological models of the thermo-physiological responses to outdoor thermal climate, but is limited in its assessment to standard conditions with a fixed moderate metabolic rate (135 W/m^2^, walking at 4 km/h on the level) and with typical clothing for urban populations that is incorporated and modelled based on actual observations from human clothing behaviour in urban environment conditions (Lindner 2010). Within the air temperature range approximately between −20 to +30 °C, the observed basic clothing insulation varies with ambient air temperature and most probably changes also with the exposure time planned by the residents in urban environments, and their physical activity levels (Havenith et al. 2012). Most likely the exposure time in those observations is shorter than a work shift. The clothing insulation is corrected for wind and body movement (Havenith et al. 2012; Havenith et al. 1999; Parsons et al. 1999). It was proposed that the clothing insulation should be in the range of 2.6 to 0.5 clo (Richards and Havenith 2007). The clothing model (in general, clothing insulation decreases with the increase of air temperature) incorporated into the UTCI is summed from local insulation values as a function of the ambient temperature in the study by Havenith et al. (2012). On the warm side at air temperature (T_a_) about 32 to 40 °C, the basic leisure clothing insulation (I_cl_) is modelled to be relatively constant at about 0.3 clo. The number of the observed clothing insulation data over ambient temperatures of higher than 30 °C is limited (Havenith et al. 2012). Furthermore, the clothing behaviour of urban residents in warm and hot environments does not always apply to occupational settings where workers wear special clothing for safety and protection.

In workplaces and in the context of global warming, the criteria of air temperatures for hot weather warnings in most of the countries are higher than 30 °C (Havenith and Fiala 2016) and the insulation of work or protective clothing in occupational settings is usually higher than 0.5 clo. Consequently, occupational heat stress is likely underestimated by the UTCI. This aspect is a further limitation of the UTCI when applied to the assessment of occupational heat stress and heat strain for an eight-hour shift in workplaces when facing heat waves. A calculation tool for UTCI is available on the website (http://utci.org/) based on a simplified version of the model of human thermoregulation. Although UCTI is based on an advanced and complex model, it is more confined to the assessment of the outdoor thermal environment in biometeorological applications (Havenith and Fiala 2016). A recent attempt has been made to extend the application of the UTCI to varying activity levels and exposure durations (Bröde et al. 2016). However, the UTCI and its incorporated clothing model suffers similar limitations as WBGT and PHS models for the assessment of heat stress when wearing protective clothing, therefore, it needs further improvements to assess and predict occupational heat stress of workers wearing protective clothing in hot environments (Havenith et al. 2012; Bröde et al. 2016).

### Future heat stress assessment in occupational settings

Current meteorological data are more adequate for the assessment of the impact of climate on population health, but less adequate for occupational health (Parsons 2013). For the assessment of occupational heat stress, the following factors needs to be taken into consideration: 1) in addition to weather data, local workplace conditions that can be altered by local heating and cooling (e.g. use of furnace and air-condoning in industries), the design and materials of buildings, shade in outdoor environments (Johansson et al. 2017; Yahia et al. in press), etc. Local climate in urban environments (e.g. urban heat island effect) is influenced by urban forms, buildings, street canyons, plants, etc., therefore the microclimate in urban environments should be directly measured or predicted, e.g. using ENVI-met model (Taleghani et al. 2015) and other methods (de Dear and Brager 1998; Yang et al. 2014; Vuckovic et al. 2016) based on meteorological data, 2) special work and protective clothing rather than ordinary clothing worn by the general population, 3) physical work intensity. These three factors usually create more heat stress in occupational groups than in the general population and lead to underestimation of heat stress using currently available heat stress indices. Thus, there is a need for suitable heat stress indices, methods and/or standards to account for the above three workplace and individual factors for reasonable occupational heat stress assessment using routine meteorological data.

Future heat stress assessment tools in the context of climate change should be valid in high-risk climate zones, interpretable and translatable, acceptable and accessible. The assessment should be linked to recommendations of flexible and hierarchical control measures by taking vulnerabilities into consideration (Spector and Sheffield 2014). It should be based on solid thermal physiological and physical basis for both hot dry and hot humid environment conditions (Fiala and Havenith 2015) and allow for a wide range of clothing adjustments including protective clothing. Epstein and Moran (2006) emphasized that an applicable heat stress index must meet the following criteria: 1) feasible and accurate at relevant range of environmental and metabolic conditions, 2) considers all six factors, 3) the measurement should reflect worker’s exposure, 4) exposure limits should be reflected by physiological and/or psychological responses. We can add that occupational heat stress indices should also make clear links to the practical consequences for the work activities, including productivity loss and economic impacts (Kjellstrom 2016).

## Protective strategies

The alleviation of occupational heat stress impacts on health and productivity of working people is ultimately through mitigating climate change. This is what is recommended by the IPCC (Smith et al. 2014) and the global climate change policy meeting in Paris in 2015 produced an agreement by 195 nations to limit the global mean temperature increase to less than 2 °C, and ideally at 1.5 °C (United Nations 2015). The current voluntary offers of greenhouse gas emission limitations would lead to a temperature change of 2.7 °C, so further policies and actions need to be developed in most countries.

Even a climate change induced global mean temperature increase of 1.5 °C will lead to more frequent and more intense hot periods in large parts of the world: “heat waves” in countries with generally cool climate and sustained intensified hot seasons in the already hot countries (Collins et al. 2013). Therefore, protection against occupational heat stress via “adaptations” to climate change will be needed around the world, and such prevention adaptation actions are already now taken in locations with very hot days. The following sections give examples of protective approaches.

### Heat wave monitoring and warning

A EuroHEAT project coordinated by the Global Change and Health programme of the WHO Regional Office for Europe and co-funded by DG SANCO in the framework of the EU Public Health Programme (http://ec.europa.eu/health/climate_change/extreme_weather/heatwaves/index_en.htm#fragment0) has provided the possibility to predict heat waves in Europe. EuroHEAT improves mainly the preparedness and responses of health systems to protect public health (rather than occupational health) from heat waves. The project developed European heat wave and hot alert systems, and heat wave probability prediction – web-based Climate Information Decision Support Tool for Heat in Europe (EuroHEAT 2006), which has already been linked to national weather forecast. National Heat Health Warning Systems (HHWSs) have, in general, lead times of between 0 and 3 days and provide deterministic heat warnings. The climate information decision support tool provides probabilistic medium-range heat information. The medium-range prediction complements the national warning system with medium-range heat forecasts. Medium range in meteorology means lead times of between 3 and 10 days. Each day a forecast of the probabilities for heat events for the following 9 days is issued (EuroHEAT 2006).

However, the above weather warning does not provide specific heat warning for industries and workplaces for more than 10 days ahead. A recently started EU Horizon 2020 – Research and Innovation Framework Programme (H2020-PHC-2015) “*Integrated inter-sector framework to increase the thermal resilience of European workers in the context of global warming (HEAT-SHIELD)*” plans to develop an online open access service anticipating and warning for events that may pose a threat specifically to the workforce. It forecasts weather patterns in different European regions for various climate change scenarios to produce detailed information on workers’ conditions in time horizons between a month and a year ahead. The project is planned to be completed by 2020.

### Local heat condition assessment via climate CHIP

Climate CHIP is a non-profit website (ClimateCHIP 2016) supported by charitable grants from organizations and individuals concerned about local and global threats to human health and society from climate change. The website aims to provide a range of information and resources about heat stress and related health impacts of climate change on working people and the general population. The website provides the possibility to input temperature and humidity data to calculate WBGT and UTCI in the shade. It uses weather station and climate modelling data from all over the world to project the trend of temperature, humidity and other climate variables for several decades in areas within 0.5 × 0.5 degree grid cells (appr. 50 × 50 km at the Equator) using several climate models.

This website is still under development and eventually new applications that take account of heat exposures outdoors in the sun and the impacts of different clothing will be included. Monthly climate condition and heat data since 1980 are included for local areas (in 67,000 grid cells over land). In addition, data from specific weather stations (more than 18,000) for daily heat analysis can be accessed via the Hothaps-Soft database and software (http://climatechip.org/hothaps-software). This data can be used by local enterprises, government departments, community organizations, schools and other interested parties to assess heat stress risks in their own locality.

### Reduce sources of heat in workplaces

The heat wave warnings outlined above can help the general public to prevent heat related illnesses. The benefit for occupational health is, however, limited due to the fact that factors like local heat sources, different workplace microclimates, endogenous heat production, protective clothing, etc. are not taken into account for the actual health effects on workers. The macro-level heat wave warning services for the general public are based on large geographical areas so they are not always suitable for warnings for individuals or organizations such as workplaces. There is a need for specific actions to cope with occupational heat stress, e.g. reduction of sources of local heat or the application of personal and microclimate cooling measures. Bearing in mind the six basic factors as described previously, corresponding measures can be taken to reduce heat stress and to increase heat loss from the body to the environment. Such measures include the reduction of radiant heat, avoidance of work in direct sunlight (working in shade) and the shielding of local industrial heat sources, increased air velocity through local ventilation using electric fans (Jay et al. 2015) within a certain range of temperature and humidity, and the reduction of ambient air temperature and humidity, e.g. using renewable energy powered air-conditioning, etc. (Grossman 2002; Balaras et al. 2007; Lundgren and Kjellstrom 2013; Lundgren-Kownacki et al. in press).

### Reduce physical work intensity

A normally functioning human body generates heat all the time even during resting to maintain body temperature at a healthy level. On average, the internal heat production i.e. metabolic rate (ISO 8996, 1989) is about

1. 65 W per m^2^ body surface area (approx. 1.8 m^2^ of an average person) during sitting and resting e.g. sedentary work,
2. 100 W/m^2^ at low physical activity e.g. light manual work, walking up to 2.5 km/h,
3. 165 W/m^2^ at moderate physical work e.g. sustained hand and arm work, walking at 2.5–5.5 km/h,
4. 230 W/m^2^ at high physical work intensities e.g. intense arm and trunk work, walking at 5.5–7 km/h and
5. 290 W/m^2^ at very high physical work e.g. intense shovelling or digging, walking at greater than 7 km/h.

The excess heat must be dissipated to the environment. When faced with external heat exposure and with barriers of heat exchange like protective clothing, heat loss from the body becomes difficult. A strategy is to reduce physical work intensity and increase more frequent and longer rest periods in order to decrease the body internal heat production (Kjellstrom et al. 2009). In the standard ISO 7243 (1989), the physical work intensity is recommended to be reduced with the increase of WBGT temperature. Depending on work load, heat stress management should start when WBGT reaches 18 °C for workers who are not acclimatized, and physical work should be stopped when WBGT is 33 °C or above for those who are acclimatized (Table 1).

**Table 1:** WBGT reference values in relation to physical work intensity and acclimatization

Work intensity	Metabolic rate (*M* in *Wm* ^−2^)	WBGT reference value (°C)	
		Acclimatized person	Not acclimatized person
Resting	*M* < 65	33	32
Low	65 < *M* < 130	30	29
Moderate	130 < *M* < 200	28	26
High	200 < *M* < 260	25 (26)*	22 (23)*
Very high	*M* > 260	23 (25)*	18 (20)*

*Values in brackets refer to sensible air movement; values without brackets refer to no sensible air movement (modified from ISO 7243; Parsons 2013; ISO 8996)

It needs to be emphasized that the heat protection via reduced work intensity may significantly reduce work productivity and economic returns both for individuals and organizations (Kjellstrom et al. 2009). This can be a major problem for individuals who get paid by work output, and the lost income for families, enterprises and communities may become a major problem due to climate change. The global assessment of this impact of occupational heat stress indicated that already in 2030 the economic loss for the world is at trillion-dollar level (DARA 2012).

### Personal protection and protective clothing

Clothing insulation and evaporative resistance hinder heat transfer through convection, radiation and evaporation from the body to the environments, leading to increased heat strain. In the presence of sunshine, the clothing should be lightly coloured and loosely fitted to the body to better reflect solar radiation and facilitate convective and evaporative heat exchange.

However, in many occupational settings, work overalls or protective clothing is needed for protection from physical, biological and chemical hazards, for instance, protective clothing for Ebola emergency workers (Kuklane et al. 2015), building and construction workers, farmers and fire-fighters. With powerful radiant heat sources, flames and hot liquid, protective clothing with several layers e.g. reflective layer and outer shell against flames, hot liquid barriers and thermal resistant layer to protect against flames, hot liquid and radiant heat are required in order to avoid burn injuries (Rossi 2014). When heat exposure is unavoidable and work has to be done, in order to still maintain work performance, personal and microclimate cooling measures may be the only way to alleviate heat strain, improve thermal comfort, protect health and maintain productivity (Wang and Gao 2014; Kuklane et al. 2015). Moreover, wearable and movable personal microclimate cooling, in contrast to air conditioning for an entire room or building, can increase energy efficiency and provide possibility of coping with heat waves in areas where air conditioning is not commonly used, such as in the Nordic countries (Gao et al. 2012).

### Cooling with ventilation and phase change materials

Air movement around the body increases convective heat loss to the environment when the air temperature is lower than the skin temperature. The average skin temperature in a thermo-neutral environment is about 34 °C, which means that in many situations, increased ventilation leads to increased heat loss from the body. The skin temperature may increase a few degrees, e.g. 1–4 °C when exposed to heat. In such situations, ventilation can still benefit evaporative cooling if air humidity has not reached saturation (i.e. relative humidity of air is less than 100%). The study on a thermal manikin with constant surface temperature (T_manikin_ = 34 °C) wearing “ventilation clothes” with integrated electric fans used in a hot environment, simulating a heat wave (T_a_ = 34 °C, RH = 60%, v_a_ = 0.4 m/s), indicated that the ventilation clothes enhanced evaporative cooling compared with nude and a normally clothed manikin (Gao et al. 2011b). The results imply that ventilation clothes can be used as a personal preventive measure in similar conditions when confronted with hot climates (Gao et al. 2011b, Zhao et al. 2013a, Fig. 1). The ventilation cooling shirt with integrated electric fans was further studied on human subjects who wore the shirts while doing office work in a climatic chamber simulating a hotter climate (T_a_ = 38 °C, RH = 45% and v_a_ = 0.4 m/s). The results showed that the cooling effect was mainly limited to torso skin. Although the thermal sensations for both the whole body and the torso were alleviated, core body temperature and mean skin temperature were not affected (Zhao et al. 2015b). In very hot environments, the cooling effect of the fans in these garments is gradually approaching its limit (Jay et al. 2015). Thus, other cooling methods are warranted (e.g. a cooling vest with phase change material), particularly when the humidity is also high, and/or when the clothing is impermeable (Gao et al. 2010; Zhao et al. 2013b; Kuklane et al. 2015).

**Fig. 1:**
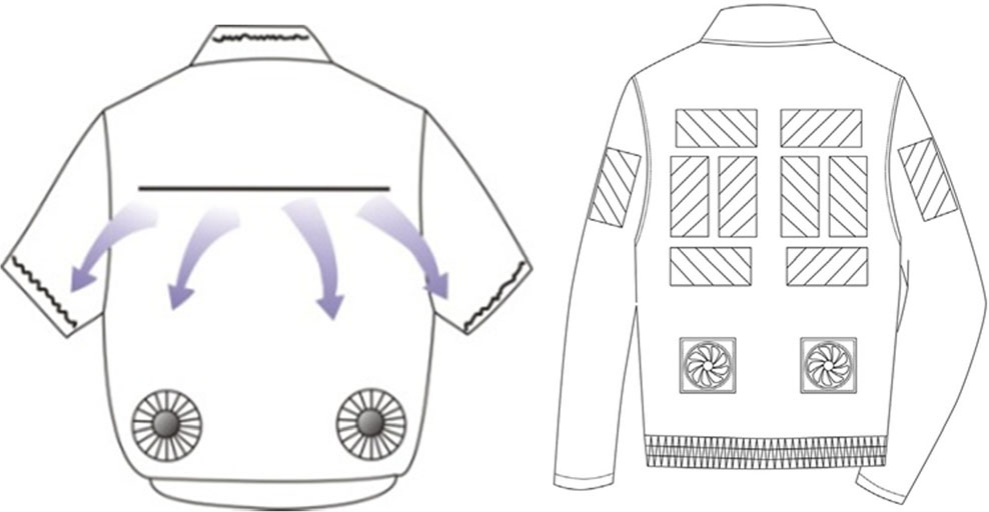
left: Ventilated clothing with integrated electric fans and opening in the back (Zhao et al. 2013a), right: hybrid personal cooling garment incorporated with PCM and ventilation fans (Lu et al. 2015)

Ventilation and evaporation combined is an effective way to utilize the body’s own sweating capacity to regulate body heat loss. Various ventilation solutions are summarized by Kuklane et al. (2012). Ventilation through clothing can be increased by the use of air permeable clothes; increasing possibilities for ventilation (i.e. design solutions); utilization of active ventilation (e.g. fans or compressed air), etc., however, in hot and polluted environments, the above methods cannot be used. Ventilation of special protective clothing (e.g. for chemical, biological, radiological and nuclear protection) does require inlet air filtering or a separate, compressed air source. When intense physical activity is carried out in an extremely high temperature environment, considerable air flow (>100 l/min) is required for sufficient cooling. The larger the ventilated skin area, the more effective is the cooling due to enhanced evaporation. Ventilation with dry air, either environmental or from a separate system, also has a stronger cooling effect due to improved evaporation.

A recent approach to thermal modelling has shown that electric fans increase the “critical air temperature limit” by approximately 3–4 °C for both young and older people (Jay et al. 2015). The use of fans as a simple and cost-effective intervention benefits body cooling up to air temperatures that exceed all of the peak outdoor conditions reported during ten of the most severe heat waves over the past 20 years. The public should therefore not be advised to stop using electric fans during any heat wave (Jay et al. 2015). However, the cooling benefit of fans decreases when the humidity increases so the use of fans alone would not provide complete protection from heat stress. In addition, conventional electric fans are usually stationary, not wearable and not able to be moved so they are more appropriate for indoor use. A recent meta-analysis of the effects of microclimate cooling systems on human performance in thermally stressful environments, has shown that natural air-cooled garment and phase change material (PCM) cooling is more applicable for the majority of outdoor workers due to the mobility requirements (Chan et al. 2015).

Phase change materials (PCMs) absorb or release “latent heat” when they change phases (e.g. from solid to liquid). The latent heat of the PCMs can be used to create cooling by absorbing heat while the PCMs are melting from solid to liquid. PCMs have been investigated for applications as building materials to store solar energy, for cooling and heating in indoor environments and for energy efficiency (Sharma et al. 2009; Zhou et al. 2012). Similarly, PCMs make thermal-regulated clothing that alleviates heat strain and improves thermal comfort (Gao 2014). A number of studies on personal cooling using PCMs showed its effectiveness in alleviating heat strain in occupational settings (Bennett et al. 1995; Choi et al. 2008; Chou et al. 2008; Reinertsen et al. 2008; Gao et al. 2011a), and during and after sports and exercises (Webster et al. 2005; House et al. 2013; Zhao et al. 2015a). However, few studies have tried to apply PCMs to improve thermal comfort from a heat wave perspective (Gao et al. 2012). Further applications of PCMs to microclimate cooling around the body for the general population and occupational groups are needed. In order to increase cooling effectiveness, factors (e.g. melting temperature, the amount of material, covering area) that affect the cooling efficiency and duration of the PCMs should be taken into consideration (Gao 2014).

Combinations of the above cooling methods can also be used, e.g. personal cooling system (PCS) incorporated with phase change materials (PCMs) and ventilation fans (Lu et al. 2015, Song and Wang 2015, Fig. 1), to maximize cooling under a variety of conditions. In hot and humid conditions, evaporative cooling of sweat would be low but PCMs would still work, while in hot and dry conditions, ventilation is of an advantage.

Ice is the most common phase change material. The latent heat of ice (335 kJ/kg) is higher than those of other types of PCMs, therefore the cooling rate is high (Gao 2014). Ice can be used in occupational settings with extremely hot environments with very high physical work and exercise intensity (i.e. overwhelming heat stress) to alleviate heat strain, increase performance and improve survivability (Smolander et al. 2004; Duffield and Marino 2007). However, the application of ice and PCMs needs logistic support.

## Conclusions and future work

Heat stress is a well-known occupational health hazard, which requires measures to protect the health and productivity of working people. As global warming continues and weather patterns become increasingly unstable, heat waves will become more frequent and intensive, and must be considered as an occupational health and public health risk in almost all settings. In tropical and sub-tropical areas with very hot seasons, the occupational heat stress problems are most prevalent.

One of the work packages in the above mentioned EU Horizon 2020 project (HEAT-SHIELD) will review the current occupational heat stress indices and propose a new improved approach that includes weather data, and is expected to overcome the limitations of current approaches. Another HEAT-SHIELD work package will analyse how different population groups are affected by heat while working, and a third package will screen and optimize technical and biophysical solutions to counter the heat-induced risk to workers’ health for key European industries in the context of global warming.

The efficiency of managing heat stress in the context of climate change relies heavily on early warning systems, valid and reliable methods to measure and assess heat exposure, and the knowledge of the efficiency and cost-effectiveness of preventive measures. This overview has shown that the body of knowledge in this area is steadily growing, and that routine data from existing weather stations is very valuable. However, due to lack of some of the important variables involved in the heat exchange between the body and environment, such as 1) local heat sources, 2) special work and protective clothing, 3) physical work intensity, the analysis needs to be complemented beyond routine weather station data, particularly for heat stress assessment in workplaces. These new developments will require interdisciplinary research including expertise in ergonomics and occupational medicine.

The development and evaluation of preventive measures depend on the quality of heat stress assessments as well as on technological innovation (e.g. new types of cooling clothing, ventilation solutions, and renewable energy cooling systems). The efficiency of different approaches needs to be evaluated, including the cost-effectiveness of such interventions. Appropriate protective strategies also need to be designed for working population with vulnerable individuals whose occupational health and performance are negatively impacted by heat stress.
